# Weekday and weekend patterns of objectively measured sitting, standing, and stepping in a sample of office-based workers: the active buildings study

**DOI:** 10.1186/s12889-014-1338-1

**Published:** 2015-01-17

**Authors:** Lee Smith, Mark Hamer, Marcella Ucci, Alexi Marmot, Benjamin Gardner, Alexia Sawyer, Jane Wardle, Abigail Fisher

**Affiliations:** Department of Epidemiology and Public Health, Health Behaviour Research Centre, University College London, London, WC1E 6BT UK; Department of Epidemiology and Public Health, Physical Activity Research Group, University College London, London, WC1E 6BT UK; Institute for Environmental Design and Engineering, The Bartlett Faculty of the Built Environment, University College London, London, NW1 2BX UK

**Keywords:** Physical activity, Sitting, Standing, Patterns, Levels, Office workers

## Abstract

**Background:**

There is a growing body of research into the total amount and patterns of sitting, standing and stepping in office-based workers and few studies using objectively measured sitting and standing. Understanding these patterns may identify daily times opportune for interventions to displace sitting with activity.

**Methods:**

A sample of office-based workers (n = 164) residing in England were fitted with thigh-worn ActivPal accelerometers and devices were worn 24 hours a day for five consecutive days, always including Saturday and Sunday and during bathing and sleeping. Daily amounts and patterns of time spent sitting, standing, stepping and step counts and frequency of sit/stand transitions, recorded by the ActivPal accelerometer, were reported.

**Results:**

Total sitting/standing time was similar on weekdays (10.6/4.1 hrs) and weekends (10.6/4.3 hrs). Total step count was also similar over weekdays (9682 ± 3872) and weekends (9518 ± 4615). The highest physical activity levels during weekdays were accrued at 0700 to 0900, 1200 to 1400, and 1700 to 1900; and during the weekend at 1000 to 1700. During the weekday the greatest amount of sitting was accrued at 0900 to 1200, 1400 to 1700, and 2000 to 2300, and on the weekend between 1800 and 2300. During the weekday the greatest amount of standing was accrued between 0700 and 1000 and 1700 and 2100, and on the weekend between 1000 and 1800. On the weekday the highest number of sit/stand transitions occurred between 0800 to 0900 and remained consistently high until 1800. On the weekend, the highest number occurred between 1000 to 1400 and 1900 to 2000.

**Conclusion:**

Office based-workers demonstrate high levels of sitting during both the working week and weekend. Interventions that target the working day and the evenings (weekday and weekend) to displace sitting with activity may offer most promise for reducing population levels of sedentary behaviour and increasing physical activity levels, in office-based workers residing in England.

## Background

Regular participation in physical activity aids the prevention of over 20 chronic conditions such as cardiovascular disease and musculoskeletal problems [1]. However, in contemporary society where physical activity levels are generally low [2], sedentary behaviour (i.e., sitting) has emerged as a key area for health [3-5]. An emerging body of literature suggests that prolonged bouts of sedentary behaviour are associated with higher risk of cardiovascular disease and mortality, even after statistical adjustment for moderate-to-vigorous physical activity (MVPA; e.g., brisk walking) [6,7]. Some data also suggest that interruptions in periods of sedentary time are beneficially associated with metabolic health [6,8].

In light of the beneficial effects of physical activity and the detrimental impact of sedentary behaviour, physical activity guidelines to maintain good health have been developed. Two of the key messages in the English guidelines for adults are (i) achieve at least 150 minutes of moderate-intensity aerobic activity every week, this can be achieved by completing 30 minutes of MVPA on 5 days of the week, and (ii) all adults should minimise the amount of time spent being sedentary for extended periods [9]. Other westernised countries have similar guidelines (see e.g., http://www.health.gov/paguidelines and http://www.health.gov.nz/our-work/preventative-health-wellness/physical-activity).

However, despite these physical activity guidelines, physical activity levels are low and sitting time high worldwide. Sedentary occupations have increased by 83% since 1950 (http://www.heart.org), and in a recent study of adults (>15 years) from 122 countries [10], approximately a third (31.1%) were physically inactive (defined as not meeting physical activity recommendations), and inactivity was higher in women than in men. Moreover, inactivity was higher in higher income countries. It is also well documented that daily physical activity levels differ between occupation types, with white-collar workers achieving the lowest levels.

There is a growing body of research on population physical activity levels using both subjective and objective tools [10,11], but there is little research on the total amount and patterns of sitting, standing and stepping in specific populations (e.g., office workers). Understanding these levels and patterns in different populations may identify at-risk groups (those with low physical activity and high sedentary time), and daily times where there is most scope to increase physical activity or reduce sedentary time. One group who may be at risk of low levels of physical activity and high levels of sedentary behaviour are office workers. Clemes et al. examined sedentary behaviour and physical activity during and outside working hours, using the Actigraph GT1M accelerometer. Participants spent the greatest proportion of the day sedentary on work and non-work days accounting for 68% and 60% of accelerometer wear time, respectively. It was also found that on workdays 71% of working hours were spent in sedentary activities [12]. Parry et al. found in a sample of office workers that sedentary time, monitored by the Actical accelerometer, accounted for 81.8% of work hours (light activity 15.3% and MVPA 2.9%) [13]. However, the Actigraph and Actical accelerometers quantify time spent in different intensities of activity by summing time above and below specified activity count thresholds. This method works reasonably well for identifying MVPA but is less accurate for distinguishing between sedentary, standing, and light activity [14]. Thus, methods that employ postural allocation may be more reliable. Recently a thigh worn accelerometer and inclinometer (ActivPal) has become available that employs this postural allocation method. Using the ActivPal, sitting time was objectively quantified in a sample of office workers and it was found that participants sat on average for approximately 66% of their working day [15]. Estimates of physical activity levels in office-based workers suggest daily step counts of no more than 4000 to 6000 [16]. Therefore, office workers may be an at risk population of low levels of physical activity and high sitting time. It seems plausible that the office may offer a platform for activity promotion and reductions in sitting time but research in this context is limited [17].

To date, there have been two reviews on workplace-based interventions to increase physical activity [18,19]. The majority of the interventions identified in the reviews have been based on bolstering workers’ motivation or capability for translating motivation into action, or offering greater physical activity opportunities to those motivated to be more active. These interventions have typically yielded small effects. Workplace-based interventions have also been carried out to reduce overall sitting time [20]. Evidence of their effectiveness has been mixed: a systematic review [20] of six workplace-based interventions targeting sitting time found none to have been effective, though more recent interventions have achieved reductions in sitting [21-27]. Until now, no study has used postural allocation techniques to understand levels and daily patterns (including weekends) of sitting, standing and stepping in office-based workers, this may allow for the development of more consistently successful interventions to increase physical activity and reduce sitting time, by identifying times that are associated with lower activity and so offer greater opportunity for intervention.

### Aim

Using data from the Active Buildings study [16] (http://www.activebuildings.co.uk), we analysed total amounts and patterns of daily sitting, standing, and stepping, in a sample of office-based workers, residing in urban areas predominantly in the Southeast of England.

## Method

Full details of participant recruitment and study procedures have been reported elsewhere [17]. In brief, Active Buildings is a cross-sectional study examining associations between office layout and stepping, standing and sitting in office-based workers (≥18 years) from 8 office buildings. Data were collected between 2013 and 2014. Participants were asked to complete questionnaires on standard demographics (age, sex, ethnicity, job role etc.). They were also fitted with the ActivPal^3^™ accelerometer (http://www.paltechnologies.com/), attached to the middle of the thigh at the workplace by trained research assistants, which was worn all day for the following five consecutive days, always including Saturday and Sunday and during sleep. Waterproof adhesive dressing was fitted over the device permitting bathing and swimming without the need for removal. Participants were provided with four additional waterproof adhesive dressings in case the original dressing needed to be replaced. Previous research has shown that three consecutive days of objective data is needed to accurately measure average daily time spent in different activity intensities (e.g. see [28,29]). At the end of the wear protocol, research assistants returned to the workplace to collect the devices. The ActivPal has been successfully used in studies of office workers and adults [30,31] and has been validated for step count, time spent sitting, standing and walking and for identifying postural transitions [32]. Explicit written informed consent was obtained from all participants. Ethical approval was obtained through the University College London Research Ethics Committee (Reference number: 4400/001).

### ActivPal processing

On completion of the monitoring wear protocol, ActivPal data were downloaded at the research centre. The ActivPal records data in 20 second Hz. Data were opened in the ActivPal interface program and exported into Excel. Time-stamped data were summarised in 15 second intervals and analysed in hourly intervals. All data collected were visually inspected for unusual episodes, none were observed. Daily times spent sitting, standing, or stepping, frequency of sit/stand transitions, and step counts, were calculated for participants with data on three or more weekdays and at least one weekend day (n = 162, 99%). Weekday times, frequencies and counts were calculated for those with three or more weekdays of data (n = 164, 100%); weekend times, frequencies and counts for those with both weekend days (n = 146, 89%). Daily data were included in the analyses only when ActivPals were worn for 24 hrs (00:00 to 00:00). Days when the ActivPal was removed for any period of time by the participant were excluded. Compliance to wearing the device was confirmed by self-report. Weekday and weekend sitting was categorised as any sedentary time accumulated between 0700 and 2300 where participants on the whole were most likely to be awake (determined by the distribution of ActivPal outcomes).

### Analysis

Characteristics of the study population were summarised using descriptive statistics. Average daily time spent sitting, standing, and stepping, and average daily step count and sit/stand transitions, as well as average length of sitting bout were calculated. This was repeated for weekdays and weekend days, and for men and women separately. Paired t-tests were then carried out to investigate differences between ActivPal outputs on weekdays and weekends, and independent t-tests between males and females. For illustrative purposes, pie graphs were generated for average proportions of weekdays and weekend days spent sitting, standing and walking between 0700 and 2300 (the “waking day”). Next, average proportions of each hour spent sitting, standing and walking, average step count, and average sit/stand transitions were calculated for weekday and weekend day times, and presented using bar graphs and stacked bar graphs.

## Results

A total of 164 participants provided valid ActivPal data and so were entered into analyses. Sixteen participants did not provide data on demographics. Of the 148 participants who provided demographic data, 55% were women, 82% were white, 15% had a managerial role and 45% had a professional role, and the average age was 39 (SD ± 10.57) years.

**Figure 1:**
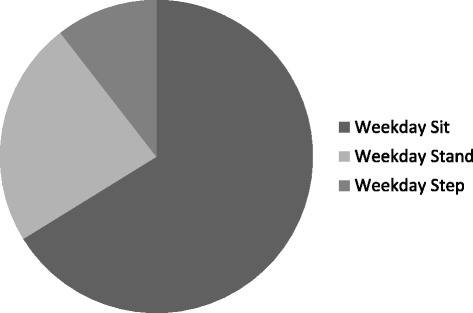
**Proportion of weekday time spent sitting, standing, stepping (0700 to 2300).**

**Figure 2:**
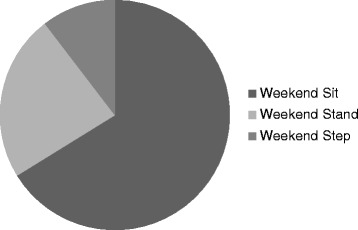
**Proportion of weekend time spent sitting, standing, stepping (0700 to 2300).**

Between the hours of 0700 and 2300 the proportion of weekdays and weekend days, on average, spent sitting (66.2% versus 66.2%), standing (23.3% versus 23.4%), and stepping (10.5% versus 10.4%) were approximately equal (see Figures 1 and 2).

Over a 24 hour period (00:00 to 00:00) participants stood for an additional 15 minutes a day on weekends. Participants took slightly more steps in total during weekdays than weekend days (9682 steps versus 9518), and made more sit/stand transitions (n = 4 more transitions; Table 1).

**Table 1:** **Mean times (hours) spent standing and sitting and mean daily step counts and sit/stand transitions**

	**Average daily mean (SD)**	**Average weekday mean (SD)**	**Average weekend day mean (SD)**	**Difference between weekday and weekend (Paired t-test; n = 146)**
**Time spent standing**	4.1 (1.4)	4.1 (1.8)	4.3 (1.7)	0.2 hours
**(24 hour period)**	N = 162	N = 164	N = 146	p = 0.308
**Time spent sitting**	10.6	10.6 (2.1)	10.6 (2.5)	0 hours
**(0700 to 2300)**	N = 162	N = 164	N = 146	p = 0.137
**Step counts**	9737 (3517)	9682 (3872)	9518 (4615)	164 steps
**(24 hour period)**	N = 162	N = 164	N = 146	p = 0.369
**Sit/stand transitions**	52.2 (13.7)	54.2 (15.1)	50.1 (16.2)	4.1 transitions
**(24 hour period)**	N = 162	N = 164	N = 146	P = 0.002

Women stood for a mean 4.09 (SD ± 1.36) hours a day and sat for a mean 10.73 (SD ± 1.65) hours a day (0700 to 2300) and achieved a mean of 10087 steps a day and 54 (SD ± 13.7) sit/stand transitions. Males stood on average for slightly longer (4.39 SD ± 1.50; p = 0.221), sat for slightly less time (10.44 SD ± 2.08; p = 0.321), and made slightly fewer steps than females (9655 SD ± 3578; p = 0.452), but achieved the exact same number of sit/stand transitions. Daily times spent sitting, standing, and stepping, and average daily step counts and sit/stand transitions for males and females were similar for weekdays and weekend days (data not shown).

**Figure 3:**
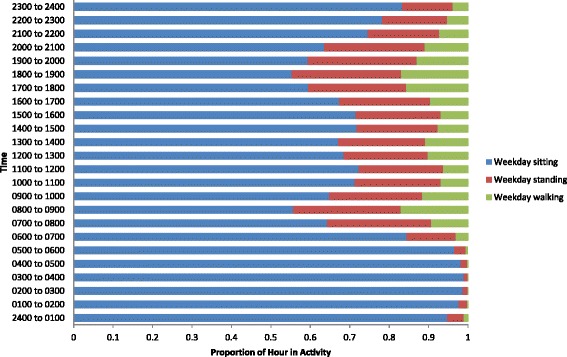
**Proportion of weekday hour spent sitting, standing and walking.**

**Figure 4:**
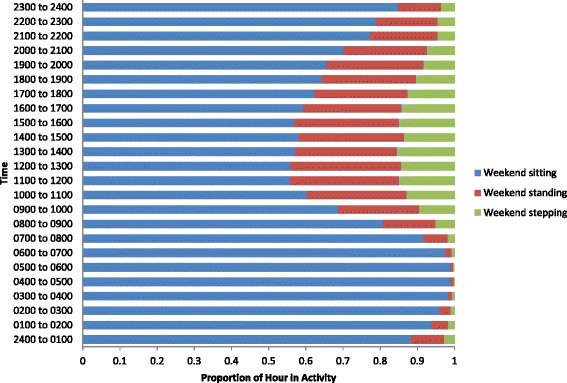
**Proportion of weekend hour spent sitting, standing and walking.**

**Figure 5:**
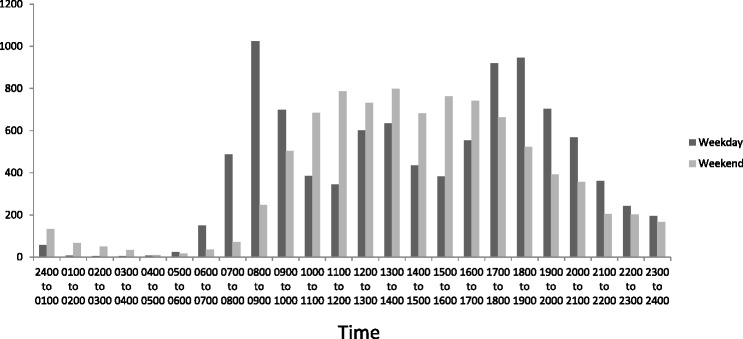
**Average step count per hour.**

**Figure 6:**
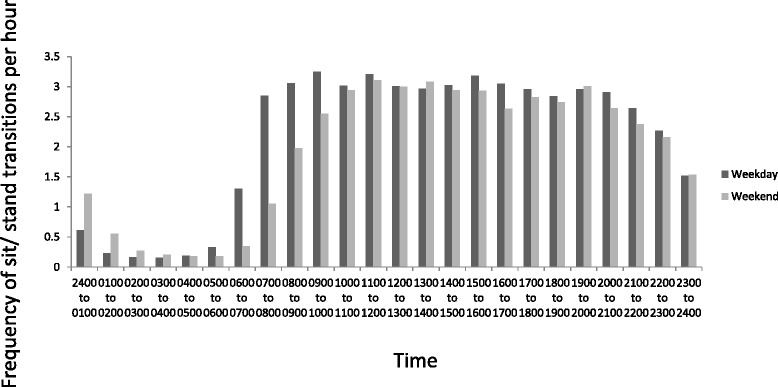
**Average sit/stand transitions per hour.**

Figure 3 shows there were peaks in weekday steps between 0700 to 0900, 1200 to 1400, and 1700 to 1900. There were no distinct peaks in weekend steps, but participants were generally active between 1000 to 1700. During the waking weekday (0700 to 2300) participants appeared to accumulate the most sitting between 0900 to 1200, 1400 to 1700, and 2000 to 2300 (Figure 4). On the weekend they appeared to sit the most between 1800 to 2300 (Figure 5). Figure 4 shows that the weekday peaks in standing occurred between 0700 to 1000 and 1700 to 2100. On the weekend the participants accumulated the most standing between 1000 to 1800 (Figure 5). On the weekday the highest number of sit/stand transitions occurred between 0800 to 0900 and remained consistently high until 1800 (Figure 6). On the weekend, the highest number of sit/stand transitions occurred between 1000 to 1400 and 1900 to 2000 (Figure 6).

**Figure 7:**
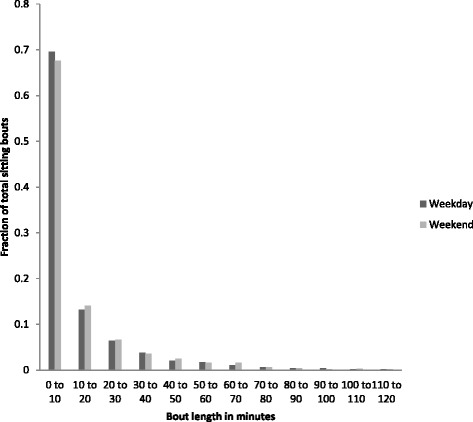
**Average length of sitting bouts.**

Figure 7 shows that the majority of sitting bouts on weekdays and weekend days on average lasted between 0 to 10 minutes, 69% and 68% of total bouts, respectively.

## Discussion

This study provides the first descriptive data on levels of objectively measured sitting, standing, sit to stand transitions, and stepping in office-based workers. The data show that participants spent the majority of their waking weekdays and weekend days sitting, followed by standing, and then stepping. Office-based workers in this study demonstrated high levels of sitting even in their discretionary leisure time (evening and weekend). This builds upon (by the use of the ActivPal accelerometer which utilises a postural allocation method to define time spent sitting, standing and stepping) and supports previous work (using various accelerometers which utilise specified activity count thresholds to define sedentary and non-sedentary time) which found that participants spent the greatest amount of time in sedentary activities on work and non-work days [12,13]. For example, Clemes et al. found using the Actigraph accelerometer that 68% and 60% of accelerometer wear time was sedentary on work and non-work days, respectively [12]. Moreover, Parry et al. found using the the Actical accelerometer that 81.8% of work hours were sedentary [13]. Tudor-Locke et al. found that daily step counts in office workers were no more than 4000 to 6000 [16]. This has important public have implications on the basis of evidence that suggests regular participation in physical activity is beneficial to health [9] and prolonged periods of sitting are detrimental [33]. Ideally these observed patterns of behaviour would be reversed so the greatest proportion of the waking day would be spent stepping, followed by standing and then sitting.

This study is also the first to explore temporal patterns of sitting, standing and stepping in daily life. Patterns of activity on weekdays differed from weekends, although total activity (step count and sitting time) did not differ. The highest numbers of weekday steps appear to be accrued between 0700 and 0900, 1200 to 1400, and 1700 to 1900. These steps were most likely accumulated during the commute to work (0700 to 0900), on lunch breaks (1200 to 1400), and on the commute home from work (1700 to 1900). The lowest weekday stepping occurred probably during office hours (1000 to 1200, 1400 to 1700) and in the evening (2000 to 2300). Highest weekend steps were generally accrued between 1000 to 1700 and the lowest between 2000 to 2300. Our data may not reveal whether periods of inactivity represent the most opportune intervention targets; an alternative interpretation is that these periods may be naturally conducive to low activity. However, the success of interventions seeking to displace sitting with standing (e.g. Alkhajah et al. [21]), or increasing walking [18], in the workplace suggests that there is scope to increase activity during these periods. Our data suggest that interventions in office-based workers to displace sitting with stepping or standing might fruitfully target the “working day” and in particular non-lunch hours. Our data imply that office-based working practice may be particularly conducive to inactivity, and one possible intervention could be to manipulate the physical office environment to encourage movement (e.g. see, Smith et al. [17]). It also seems important to target the evening on weekdays and weekends to increase step counts. It is likely that a low number of steps were accrued in the evening as participants are taking part in sedentary activities, such as television viewing. One potential intervention could be to encourage participants to step during television commercial breaks. One recent study intervened by displacing sitting during commercials with stepping. One hour of TV commercial stepping resulted in an average of 25.2 ± 2.6 minutes of physical activity and 2111 ± 253 steps [34].

Weekday standing was at its highest during the morning (0700 to 1000) and evening (1700 to 2100), whereas weekend standing was consistent from morning through to early evening (1000 to 1800). Weekday standing was at its lowest during the “working day” (0900 to 1600) and weekend standing lowest in the evening (2000 to 2300). Weekday sitting was at its highest during the “working day” (0900 to 1200 and 1400 to 1700) and evenings (2000 to 2300) and weekend sitting in the evening (1800 to 2300). Despite the observed differences in the patterns, the total amount of sitting/standing was similar across weekdays and weekends, which suggests the participants did not modify their behaviour even in their discretionary leisure time. One possible intervention to displace sitting with standing during the “working day” could be to replace traditional sitting workstations with sit-stand workstations. In a recent study that replaced sitting workstations with sit-stand workstations, sitting time was reduced by 137 min/day [21]. Given the detrimental effects of sitting it may be beneficial for population health to displace sitting with any activity (e.g., standing [35]). Moreover, if population levels can be shifted from sitting to the lowest physical activity category (standing) consequent interventions targeting stepping may then be more successful as it reflects a more natural shift along the physical activity continuum. However, experimental research needs to be carried out on the health benefits of displacing sitting with standing before recommendations for intervention should be made. The data also suggest that a high level of sitting was accrued during the evening; interventions that encourage the displacement of sitting with activity in the evening may be beneficial.

We also investigated daily patterns of sit/stand transitions - a novel area with little prior research. The greatest number of sit/stand transitions were accrued during the “working day” on weekdays, which is logical because this is when the greatest amount of sitting occurred, and midday on weekends. In a laboratory-controlled trial conducted over an 8 h period, interrupting sitting time every 20 min with short 2 min bouts of light-intensity or moderate-intensity walking was shown to lower postprandial glucose and insulin levels in overweight/obese adults [8]. Increasing the frequency of sit/stand transitions during bouts of prolonged sitting (i.e., during the working day) may be beneficial for metabolic health [6]. However, in the present study periods of prolonged uninterrupted sitting of >20 min were rare on both weekdays and weekend days.

Interestingly, in this sample of office-based workers a high level of steps was achieved (mean of 9737 steps) just below the recommended physical activity guideline of 10,000 steps a day. This contrasts with previous data which has found on average office workers achieve between 4000 to 6000 steps a day [15]. This may be because the majority of our sample resided in London and were “young” professional adults (mean age 39 years). Young professionals in London may be more active than those residing in other locations; for example, one participating office was not accessible by private motorised vehicles, so employees had to commute to work by public transport, walking, cycling, or some combination thereof. In support, in the present sample the highest weekday step counts were observed between 0700 to 0900 and 1700 to 1900, likely when participants were commuting to and from work. Further research is needed to explore daily patterns and levels of sitting, standing, and stepping in other populations, cities and countries.

A strength of this study is the objective measure of sitting, standing and stepping employed in a sample of English office-based workers. The ActivPal accelerometer/inclinometer device classifies an individual’s free-living activity into periods spent sitting, standing, and stepping, it also records transition from sitting to standing (i.e. interruptions in sedentary time) which it has been validated for. The ActivPal’s inclinometer and unique positioning on the thigh allows the device to distinguish between different postures. The relatively small sample size of office-based employees predominately residing in London limit the representativeness of the findings. Participants sleep and wake times were not recorded, therefore, some sleep data may have been coded as sitting time and vice versa. This may have introduced error into the analysis, therefore, better methods to distinguish sleep and wake times are needed in this field of research and future studies should record sleep and wake times using participant diaries.

## Conclusion

Office based workers demonstrate high levels of sitting during both the working week and weekend. Interventions that target the working day and the evenings (weekday and weekend) to displace sitting with activity may offer most promise for reducing population levels of sedentary behaviour and increasing physical activity levels, in office-based workers residing in England.
